# Editorial: Emotional intelligence: Current research and future perspectives on mental health and individual differences

**DOI:** 10.3389/fpsyg.2022.1049431

**Published:** 2022-10-13

**Authors:** Giacomo Mancini, Roberta Biolcati, Dana Joseph, Elena Trombini, Federica Andrei

**Affiliations:** ^1^Giovanni Maria Bertin Department of Education Studies, University of Bologna, Bologna, Italy; ^2^Department of Management, College of Business, University of Central Florida, Orlando, FL, United States; ^3^Department of Psychology, University of Bologna, Bologna, Italy

**Keywords:** emotional intelligence, mental health, psychological wellbeing, individual differences, emotions

The last two decades have seen a steadily growing interest in emotional intelligence (EI) research and its applications. As a side effect of this boom in research activity, a flood of conceptualizations and measures of EI have been introduced. Consequently, the label “EI” has been used for a wide array of (often conflicting) models and measures, which has impeded consistent summaries of empirical evidence. This confusion among models/measures is problematic because different measurement approaches produce different results, which makes it difficult to theorize what EI really is or what it predicts since there is limited consistency in the empirical data. On one side there are proponents of the ability model (see Mayer et al., 2016) which recognizes that EI includes four distinct types of ability and defines EI as the ability to perceive and integrate emotion to facilitate thoughts, understand and regulate emotions to promote personal growth (Mayer and Salovey, 1997). This kind of EI would only be measurable through maximum performance tests. On the opposite side, we find supporters of the trait model. In particular, Petrides et al. (2007) defines trait EI as a constellation of emotional perceptions assessed through questionnaires and rating scales. The theory of trait EI is summarized with applications from the domains of clinical, educational, and organizational psychology (Petrides et al., 2016) and it's clearly distinguished from the notion of EI as a cognitive ability. Of course, there is no scarcity of other models and perspectives of EI, including mixed approaches, often used in professional setting to train and evaluate management potential and skills, that consider EI as a broad concept that includes (among others) motivations, interpersonal and intrapersonal abilities, empathy, personality factors and wellbeing (see Mayer et al., 2008).

In accordance with Hughes and Evans (2018), we argue that various conceptualizations of EI may be considered constituents of existing perspectives of cognitive ability (ability EI), personality (trait EI), emotion regulation (EI competencies), and emotional awareness (the aptitude to conceptualize and describe one's own emotions and those of others). Across all models, EI involves handling emotions and putting them at the disposal of thinking activity. Although EI is an ability to understand and control emotions in general, this is only a small part of some models of EI. Indeed, trait EI concerns our perceptions of our emotional world and comprises a broad collection of traits linked to the opportunity of understanding, managing, and utilizing our own and other people's emotions, helping us figure out and dealing with emotional and social situations. All these facets are critical for intelligent behavior because they enable and facilitate our capacities for resilience, communication, and reasoning, to name a few, across the life span. Indeed, existing literature suggests that individual differences in EI consistently predict human behavior and EI is now recognized by the scientific community as a relevant psychological factor for several important real-life domains, including a successful socialization, community mental health and individual wellbeing. To advance the field both theoretically and practically, this special issue aims to provide new data which may help to critically review EI's theory.

The collection of articles is quite diverse and covers a number of issues relevant to an advancement of the field by including participants from several cultural contexts (e.g., Italian, Brazilian, and Turkish). Seven articles used self-report tools for the assessment of EI, while only two studies employed an ability measure (the Mayer–Salovey–Caruso Emotional Intelligence Test). With regards to the topic being addressed, one study focuses on psychometrics, and confirms the validity of the Trait EI Questionnaire as an assessment tool for trait EI in a large Brazilian sample (Zuanazzi et al.), while seven analyzed the relationship between EI and research questions pertaining the domain of psychological health and wellbeing. Among these, the papers by García-Martínez et al. and Kökçam et al. analyze instead the relationship between EI and stress management in university students. García-Martínez et al. found mixed results compared to existing literature on the path from EI and academic achievement, while Kökçam et al. found that EI plays an important role in the identification of stress profiles.

Through a systematic review Pérez-Fernández et al. highlights that EI may be a protective factor of emotional disorders in general population and offers a starting point for a theoretical and practical understanding of the role played by EI in the management of diabetes. Along these lines Sergi et al. showed that the domains of EI involved in emotion recognition and control in the social context to reduce the risk to be affected by depression and anxiety, while Pulido-Martos et al. show the contribution of socioemotional resources (including EI) to the preservation of mental health. Iqbal et al. considered the associations among EI, relational engagement (RE) and cognitive outcomes (COs) and found that EI directly and indirectly influenced COs during the pandemic: the students with higher levels of EI and RE may achieve better COs.

Last, the two articles using the Mayer–Salovey–Caruso Emotional Intelligence Test show that coping strategies mediate the relationships of ability EI with both well- and ill-being (MacCann et al.), and give some preliminary evidence on the associations between ability EI, attachment security, and reflective functioning (Rosso).

Despite their specific aims, these studies demonstrate the importance to stake on individuals' EI to favor a high psychological and physical wellbeing. At the same time, the present articles collection highlights some open issues to be addressed by future research, including: putting order and possibly connecting the existence of many conflicting models and related measures of EI; deepen the study of the relation between EI with other partially overlapping constructs; identify the most helpful training to increase EI in individuals of all ages, such as children and their parents, adolescents, adults.

## Author contributions

GM and FA drafted the editorial. RB, DJ, and ET participated in the discussion on the ideas presented and have edited and supervised the editorial. All authors approved the submitted version.

## Conflict of interest

The authors declare that the research was conducted in the absence of any commercial or financial relationships that could be construed as a potential conflict of interest.

## Publisher's note

All claims expressed in this article are solely those of the authors and do not necessarily represent those of their affiliated organizations, or those of the publisher, the editors and the reviewers. Any product that may be evaluated in this article, or claim that may be made by its manufacturer, is not guaranteed or endorsed by the publisher.
